# Positive Heparin/PF4 Antibodies and High Mortality Rate: a Retrospective Case-Series Analysis

**DOI:** 10.21470/1678-9741-2019-0360

**Published:** 2020

**Authors:** Mehmet Ezelsoy, Kemal Tolga Saracoglu, Kerem Oral, Ayten Saracoglu, Belhan Akpinar

**Affiliations:** 1 Department of Cardiovascular Surgery, Istanbul Demiroglu Bilim University Medical School, Istanbul, Turkey.; 2 Department of Anesthesiology and Intensive Care, Health Sciences University Medical School, Istanbul, Turkey.; 3 Department of Anesthesiology and Intensive Care, Istanbul Marmara University Medical School, Istanbul, Turkey.

**Keywords:** Thrombocytopenia, Heparin-adverse effects, Cardiopulmonary Bypass, Aortic Valve, Incidence, Cause of Death

## Abstract

**Introduction:**

Heparin-induced thrombocytopenia (HIT) is a potentially lethal complication of unfractionated or low-molecular weight heparin therapy. We aimed to determine the incidence and mortality rate of patients with positive heparin/platelet factor 4 (PF4) antibodies, which is a rapid detection test of HIT.

**Methods:**

Coronary artery bypass grafting and mitral and aortic valve surgeries were evaluated. Cardiopulmonary bypass was employed in all patients. The diagnosis of HIT was based on immunological assays. Postoperative complications, mortality rates, and the causes of death were specified in patients with positive heparin/PF4 antibodies.

**Results:**

Postoperative thrombocytopenia was detected in 257 patients. Twenty of these patients undergoing open heart surgery were included in the final analysis. Antibodies against heparin/PF4 complex were positive in 20 patients. The mean body mass index was 28.8±2.3 kg/m^2^, mean value of left ventricular ejection fraction was 48.3±6.7%, cardiopulmonary bypass time was 113.0±35.0 min, aortic cross-clamping time was 88.0±32.7 min, mean intensive care unit length of stay was 10.9±4.9 days, mean preoperative platelet count was 307.250±88528 platelets/microliter, and mean postoperative platelet count was 243.050±89.354 platelets/microliter. The mean duration of heparin exposure was 6.9±2.9 days. The mortality rate was 45% (nine patients) and 1.2% (three patients) in heparin/PF4 complex positive and negative patients, respectively.

**Conclusion:**

Although the incidence of HIT was low in patients undergoing open heart surgery, an increased rate of early mortality was observed in patients with positive heparin/PF4 antibodies.

**Table:** 

Abbreviations, acronyms & symbols	
BMI	= Body mass index
CPB	= Cardiopulmonary bypass
EF	= Ejection fraction
FFP	= Fresh frozen plasma
HIT	= Heparin-induced thrombocytopenia
ICU	= Intensive care unit
LMWH	= Low-molecular weight heparin
MOF	= Multiple organ failure
PF4	= Platelet factor 4
PLT	= Platelet
RBC	= Red blood cell
SD	= Standard deviation
UFH	= Unfractionated heparin

## INTRODUCTION

The management of the cardiac patients suspected of having heparin-induced thrombocytopenia (HIT) may be challenging due to a number of reasons. The functional assay which is used to diagnose the disorder is not only expensive, but also time-consuming. However, heparin should be urgently discontinued in such patients. It is also necessary to decide within a few hours whether an alternative anticoagulant agent will be started or not. Whereas delayed discontinuation of heparin can be life-threatening for patients with HIT, several risks may arise from the use of alternative anticoagulants in patients without HIT^[1]^.

Related with the duration of heparin exposure, HIT occurs in about 0.1-5% of patients^[2]^. The development of HIT carries greater risks in patients who require cardiac surgery compared to other patient populations. It has been documented that the risk for postoperative pneumonia or acute renal injury is twofold higher in patients requiring cardiac surgery, whereas the risk for thromboembolic complications is several times higher^[3-5]^. Because of a hypercoagulable state, the mortality rate may rise up to 5%-10%^[6]^. Although the risk of arterial thrombosis has been reported to be greater than the risk of venous thrombosis in cardiac surgery patients, unfractionated heparin (UFH) is associated with a five to 10 times higher risk of HIT compared to low-molecular weight heparin (LMWH)^[7]^. In addition, HIT generates nearly a 50% higher risk for in-hospital deaths in surgical patients^[8]^.

The aim of this study was to determine the rate of mortality and morbidity and the incidence of patients with positive heparin/platelet factor 4 (PF4) antibodies undergoing open heart surgery in early postoperative period.

## METHODS

**Fig. 1:**
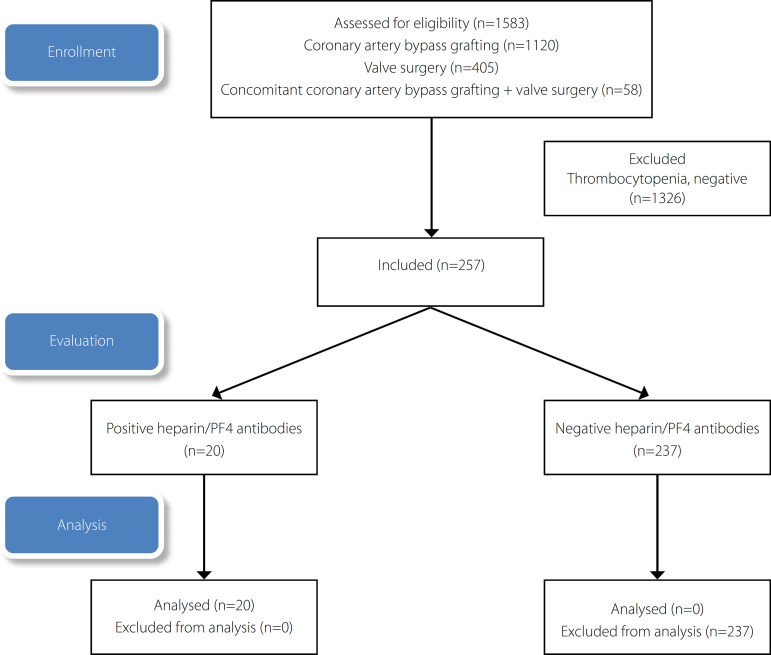
Flow diagram of the study. PF4=platelet factor 4.

Patients undergoing elective cardiac surgery between January 2014 and December 2016 were included. The data of 1583 patients were assessed for eligibility. Valve surgery and coronary artery bypass grafting were evaluated (Figure 1). Cardiopulmonary bypass (CPB) was employed in all patients. HIT was considered when all the following criteria met: (1) a platelet count was defined as: either a drop of 50% from the initial level or an absolute count of < 80.000 mm^3^; (2) no other apparent cause of thrombocytopenia; (3) positive anti-PF4 antibodies; and (4) recovery of the platelet count after the discontinuation of heparin treatment.

Clinical suspicion of HIT was based on postoperative thrombocytopenia, positive anti-PF4 antibodies, and/or the occurrence of a thrombotic event. The other possible causes of thrombocytopenia were excluded (*e.g*., intra-aortic balloon pump, severe disseminated intravascular coagulation, and non-heparin medication). Based on this clinical suspicion, anti-PF4 antibodies were then systematically determined. An alternative anticoagulant therapy of fondaparinux (Arixtra^®^) was started, and UFH or LMWH was stopped.

The variables including the duration of heparin therapy, CPB time, aortic cross-clamping time, duration of postoperative mechanical ventilation, and intensive care unit length of stay were determined and the Spearman’s correlation analysis was performed to determine whether or not these variables were correlated with each other. Furthermore, postoperative complications, mortality rates, and the causes of death were specified. Potential correlations between the exposure to heparin and a number of variables, including transfusion volumes of red blood cell concentrates, volume of fresh frozen plasma, and platelet volume, were investigated.

The study was performed in accordance with the Helsinki Declaration and approved by the Ethics Committee of Istanbul Demiroglu Bilim University. In addition, written informed consent was obtained from all study patients.

### Statistical Analysis

Descriptive statistics were summarized as mean, median, minimum, maximum, frequency, ratio, and standard deviation. The distribution of the variables was analyzed using the Kolmogorov-Smirnov test. The Mann-Whitney U test was used to investigate qualitative data. In addition, repeated measurements were analyzed using the Wilcoxon test. The Spearman’s correlation analysis was used to examine correlation between the variables. Study data was processed using the IBM Corp. Released 2013, IBM SPSS Statistics for Windows, Version 22.0, Armonk, NY: IBM Corp.

## RESULTS

The data of 1583 patients were analyzed. Postoperative thrombocytopenia was detected in 257 patients who underwent open heart surgery. Antibodies against heparin/PF4 complex were positive in 20 patients. The mean ejection fraction was 47.5% (48.3±6.7%), mean CPB time was 110 min (113.0±35.0 min), mean cross-clamping time was 82.5 min (88.0±32.7 min), and mean intensive care unit length of stay was 10 days (10.9±4.9 days).

We compared the demographic characteristics of PF4+ and PF4- patients. There were no significant differences between the patients regarding their demographic characteristics and ejection fraction values (Table 1). The duration of mechanical ventilation was significantly longer in PF4+ patients: 9.95±5.23 hours *vs*. 6.59±1.41 hours in PF4+ and PF4- patients, respectively (*P*=0.001, Table 2). The ıntensive care unit length of stay was significantly longer in PF4+ patients: 10.85±4.92 days *vs*. 1.24±0.42 days in PF4+ and PF4- patients, respectively (*P*=0.001, Table 2). The CPB time and the cross-clamping time did not differ between patients.

**Table 1:** Comparison between demographics of PF4+ and PF4- patients.

		PF4+ (n=20)	PF4- (n=237)	*P*-value
Sex	Female	11 (55%)	75 (31.6%)	0.039*
	Male	9 (45%)	162 (68.4%)	
Age (years)		67.55±5.84 (66.5)	63.9±11.6 (65)	0.171
BMI		28.8±2.31 (28.2)	26.56±1.89 (26)	0.001*
EF		48.3±6.69 (47.5)	50.38±4.85 (50)	0.090

* *P*<0.05 is the statistically significant value between groups

BMI=body mass ındex; EF=ejection fraction; PF4=platelet factor 4

**Table 2:** Comparison between outcomes of PF4+ and PF4- patients.

	PF4+ (n=20)	PF4- (n=237)	*P*-value
Cardiopulmonary bypass time (min)	113.0±35.0 (110)	107.51±46.16 (90)	0.275
Cross-clamping time (min)	88.0±32.74 (82.5)	80.92±33.38 (80)	0.349
Duration of mechanical ventilation (h)	9.95±5.23 (9.5)	6.59±1.41 (6)	0.001*
Intensive care unit length of stay (days)	10.85±4.92 (10)	1.24±0.42 (1)	0.001*

* *P*<0.05 is the statistically significant value between groups

PF4=platelet factor 4

The amount of decrease in platelet was significantly higher in PF4+ patients: 246±91.69/mm^3^ *vs*. 65.24±40.99/mm^3^ in PF4+ and PF4- patients, respectively (*P*=0.001, Table 3). The consumption rate of erythrocytes suspension was not different in groups. Perioperative fresh frozen plasma transfusion amount was significantly higher in PF4+ patients (6.9±4.19 units,*P*=0.001, Table 3). Perioperative platelet transfusion rate was also higher in this group (6.8±4.87).

**Table 3:** Comparison between blood product consumption of PF4+ and PF4- patients.

	PF4+ (n=20)	PF4- (n=237)	*P*-value
Preoperative platelet count (per mm^3^)	307.25±88.53 (300)	235.89±67.49 (226)	0.001*
Amount of decrease in platelet (per mm^3^)	246±91.69 (233)	65.24±40.99 (61)	0.001*
Perioperative erythrocytes suspension (units)	3.75±2.69 (2.5)	2.69±2.39 (2)	0.242
Perioperative fresh frozen plasma transfusion (units)	6.9±4.19 (6)	1.78±1.77 (2)	0.001*
Perioperative platelet transfusion (units)	6.8±4.87 (8)	0.78±2.98 (0.0)	0.001*

* *P*<0.05 is the statistically significant value between groups

PF4=platelet factor 4

Among PF4+ patients, a total of eleven patients developed complications (Table 4). Out of these eleven patients, four patients developed atrial fibrillation, and thus, received heparin therapy. Furthermore, two patients had hypoxia while another two patients developed multiple organ failure (MOF). Refractory hypotension and intra-aortic balloon pump placement, sepsis, and cardiac arrest were other complications observed in this study.

**Table 4:** Demographics and other outcomes of PF4+ patients.

Parameters		Min-max	Median	Mean±SD/n-%
Age (years)		57-77	66.5	67.6±5.8
Sex	Female			11 (55.0%)
	Male			9 (45.0%)
Body mass index		25-32.4	28.2	28.8±2.3
Ejection fraction (%)		39-60	47.5	48.3±6.7
Duration of cardiopulmonary bypass (min)		75-180	110	113.0±35.0
Duration of aortic cross-clamping (min)		50-155	82.5	88.0±32.7
Duration of mechanical ventilation (days)		1-25	9.5	10.0±5.2
ICU length of stay (days)		5-25	10	10.9±4.9
Preoperative blood product				
Erythrocytes suspension (units)		0-6	2	2.2±1.8
Fresh frozen plasma (units)		0-8	2	2.7±3.1
Platelet (random units)		0-8	0	2.1±3.5
Postoperative blood product				
Erythrocytes suspension (units)		0-4	1	1.5±1.7
Fresh frozen plasma (units)		0-8	4	4.0±2.1
Platelet concentrates (random donor)		0-16	7	5.9±4.5
Platelet count (per mm^3^)				
Preoperative period		165000-485000	300000	307250±88528
Postoperative period		98000-410000	233000	243050±89354
Amount of decrease		43000-85000	64500	64200±12177
Heparin exposure (days)		3-13	7	6.9±2.9
Complication	No			9 (45.0%)
	Yes			11 (55.0%)
Status	Lived			11 (55.0%)
	Died			9 (45.0%)

ICU=intensive care unit; PF4=platelet factor 4; SD=standard deviation

Out of 20 patients with positive heparin/PF4 antibodies, nine patients (45%) died. The causes of death were cardiac failure in two patients, hypotensive shock in two patients, and postoperative bleeding in another two patients. Hypoxemia, sepsis, and MOF were identified as the other causes that contributed to mortality.

In patients with positive heparin/PF4 antibodies, platelet counts significantly decreased during the postoperative period, compared to the preoperative period (*P*>0.05, Table 5). None of the patients developed venous or arterial thrombosis.

**Table 5:** Preoperative and postoperative platelet count in patients with positive heparin/PF4 antibodies.

Platelet concentration (per mm^3^)	Min-max	Median	Mean±SD	*P*-value
Preoperative	165000-485000	300000	307250±88528	0.000
Postoperative	98000-410000	233000	243050±89354	

Wilcoxon test PF4=platelet factor 4; SD=standard deviation

There were no significant differences between females and males regarding their heparin exposure (*P*>0.05). Moreover, the duration of heparin exposure did not show a significant difference between the patients who died and who stayed alive (*P*>0.05). In addition, there was no significant difference between the patients who developed complications and who did not regarding their heparin exposure (*P*>0.05, Table 6).

**Table 6:** Factors related with heparin exposure.

Heparin exposure (days)		Min-max	Median	Mean±SD	*P*-value
Sex	Female	4-13	6	6.5±2.7	0.444
	Male	3-12	8	7.3±3.2	
Status	Lived	3-10	6	6.0±2.0	0.206
	Died	3-13	8	7.9±3.5	
Complication	No	3-13	8	7.9±3.5	0.206
	Yes	3-10	6	6.0±2.0	

Mann-Whitney U test SD=standard deviation

There was no significant (*P*>0.05) correlation between the duration of heparin exposure and age, body mass index, CPB time, cross-clamping time, duration of mechanical ventilation, and intensive care unit length of stay (Table 7).

**Table 7:** Correlation between duration of heparin treatment and other factors.

		Age (years)	BMI	EF (%)	Duration of CPB (min)	Duration of cross-clamping (min)	Duration of mechanical ventilation (days)	ICU length of stay (days)
Heparin exposure (days)	r	0.268	0.294	0.244	0.283	0.355	-0.004	0.000
	p	0.253	0.209	0.300	0.227	0.124	0.987	0.999

Spearman's correlation BMI=body mass index; CPB=cardiopulmonary bypass; EF=ejection fraction; ICU=intensive care unit

Similarly, no significant correlations were found between the duration of heparin exposure and preoperative or postoperative use of blood products (*P*>0.05, Table 8). Also, there were no significant correlations between the duration of heparin exposure and the pre/postoperative platelet counts and the alteration in these values (*P*>0.05, Table 9).

**Table 8:** Relation between heparin exposure and use of preoperative and postoperative blood.

Use of blood products							
		Preoperative RBC	Preoperative FFP	Preoperative PLT	Postoperative RBC	Postoperative FFP	Postoperative PLT
Heparin exposure (days)	r	0.377	0.306	0.316	0.038	0.087	0.035
	p	0.101	0.190	0.174	0.875	0.716	0.884

FFP=fresh frozen plasma; PLT=platelet; RBC=red blood cell

**Table 9:** Correlation between heparin exposure and pre and postoperative platelet count.

		Preoperative PLT	Postoperative PLT	PLT decrease
Heparin exposure (days)	r	0.040	0.050	0.021
	p	0.868	0.833	0.929

PLT=platelet

## DISCUSSION

In this study, the overall mortality in patients with positive heparin/PF4 antibodies undergoing cardiac surgery at our medical center was 45% (n=20). The risk for this complication was also high in patients who had been in the low-risk group during the preoperative period and tested positive for PF4 antigen in the postoperative period.

The diagnosis of HIT may be challenging in patients undergoing open heart surgery. Patients undergoing CPB surgery receive large volumes of heparin infusion during this procedure, and their blood platelet counts can fall 30-50% within the first 72 hours of the postoperative period^[9]^. Thrombocytopenia was also reported to occur following transcatheter valve-in-valve ımplantation and aortic valve replacement^[10,11]^. Besides drug-induced thrombocytopenia, an extended platelet contact with artificial surfaces as a part of extracorporeal circulation is one of the major factors leading to low platelet counts^[12,13]^. For this reason, patients receiving oral anticoagulants were excluded from our study. Within the scope of the study, platelet counts were performed in all patients and a heparin/PF4 antibody test was performed in patients whose platelet counts were decreased significantly in the postoperative period. Patrick et al.^[14]^ constructed a decision tree for the management of patients with HIT. They concluded that, in all thrombocytopenic critical care patients, the routine testing is not cost-effective. However, in the presence of risk factors, PF4 testing becomes reasonable.

In addition, blood platelet counts were repeated in the 7^th^ postoperative day, since a secondary fall of ≥ 50% in platelet counts that might occur between the 5^th^ and 10^th^ postoperative days has been reported to be a potential predictor of HIT^[15,16]^.

The risk for recurrent HIT appears to be high among patients who were previously diagnosed with HIT, and therefore, the use of heparin should be avoided in these patients. Nevertheless, there are only a few studies addressing the issue with small sample size, which forms the major limitation for drawing conclusions. If possible, patients who have a prior diagnosis of HIT should delay the surgery until they become negative for HIT antibodies. Relevant guidelines recommend the use of UFH over other anticoagulants during cardiac surgery for patients with a history of HIT who have become antibody-negative within the following 100 days. Preoperative and postoperative anticoagulation, on the other hand, should be provided with an agent other than UFH or LMWH. There are studies reporting the use of lepirudin, bivalirudin, and danaparoid as alternative anticoagulant agents in cardiac surgery^[17,18]^. Whereas prophylactic use of danaparoid is not recommended in HIT patients, it can be administered at therapeutic doses. Also, therapeutic-dose fondaparinux is an acceptable alternative anticoagulant. The 2012 American College of Chest Physicians, or ACCP, guideline recommends the use of bivalirudin as an alternative anticoagulant for the cases of HIT where cardiac surgery - whether urgent or not - is required^[19]^. Nevertheless, the surgical team must be informed that the administration of bivalirudin causes coagulation of the blood accumulated in specific areas, such as pericardial sac. In patients with normal renal functions or abnormal hepatic functions, however, danaparoid or fondaparinux should be preferred (Level 2C). Argatroban, bivalirudin, danaparoid, and lepirudin are the anticoagulants commonly used in patients with HIT. In this study, we discontinued heparin in the patients who had positive heparin/PF4 antibodies during the postoperative period and we started fondaparinux (Arixtra^®^) therapy. Fondaparinux increases the risk of bleeding and has a potential risk of causing epidural or spinal hematoma in patients undergoing neuraxial anesthesia. Fondaparinux has an anti-factor Xa activity and a prolonged activated partial thromboplastin time may occur at higher doses. However, postoperative bleeding resulted only in two patients out of 20 PF4(+) patients. Therefore, we could not explain this poor outcome just with the use of fondaparinux.

Guidelines recommend platelet count monitoring from the 4^th^ to the 14^th^ postoperative day in patients with a history of CPB who receive LMWH. While HIT is more prevalent among surgical patients as compared to medical patients, a two times higher incidence has been noted in female patients^[20]^. However, we did not find a significant difference between female and male patients regarding the incidence of positive heparin/PF4 antibodies. In addition, HIT appears to be rare in patients aged < 40 years and a higher risk of HIT has been reported in older patient populations^[21]^. The average age of the patients with positive heparin/PF4 antibodies in our study was 66.5 years, which supports the evidences found in the literature.

Autoimmune anti-PF4/heparin antibodies developed in HIT activate platelets and may ultimately lead to arterial or venous thrombosis. The reported mortality rate for thrombosis is nearly 20%^[22]^. However, various studies have reported different rates. In a study involving patients undergoing valvular heart surgery, the mortality rate was 1% in patients with HIT^[23]^. Pathak R et al.^[24]^ noted that the risk ratio for the development of HIT after cardiac surgery was 0.53% and they stated that HIT was more common among female cardiac surgery patients. Moreover, the authors found a mortality rate of 9.63% in patients with HIT and the rate of patients with HIT accompanied by thrombosis was found to be 12.28%; however, the mortality rate among patients without HIT was only 2.19%.

Although none of our patients had a venous or arterial thrombosis, the overall mortality in 20 patients with positive anti-PF4/heparin antibodies was found to be 45%. The reason might be that we did not wait the development of thrombosis for the diagnosis of HIT and stopped heparin exposures as soon as an early suspicion of HIT occured. After the discontinuation of heparin therapy, we immediately started fondaparinux which, in our opinion, had a role in the prevention of thrombosis. Nevertheless, hypoxemia, MOF, sepsis, cardiac arrest, and postoperative bleeding were among the etiological and contributing factors of mortality, in our study. Even though thrombosis is the leading cause of death, this study indicated that factors other than thrombosis, such as postoperative bleeding, can lead to mortality, as well.

HIT is a rare but life-threatening complication in patients undergoing cardiac surgery. A high mortality rate has been reported in patients with HIT due to the development of venous or arterial thrombosis. However, it is possible to prevent thrombosis with early diagnosis. Particularly, platelet counts after the 5^th^ postoperative day can be predictive even if the patient is asymptomatic. There are several other factors which can potentially contribute to mortality in HIT and which can be detected in the earlier stages. We need further studies with larger sample sizes to investigate these factors.

Patients undergoing cardiac surgery or CPB have an incidence of 20% to 50% of anti-PF4/heparin antibody formation^[25]^. In our study, we declared an incidence of 8% of anti-PF4/heparin antibody formation in thrombocytopenic surgical patients. Although the total count of PF4+ patients were low, the overall mortality rate was high. This result was unique for our study and revealed that the mortality rate increases significantly in patients with thrombocytopenia and positive PF4.

Limitations

Our analysis was based on thrombocytopenia as a quantitative value. We did not evaluate the duration of thrombocytopenia which can be an associate risk factor for HIT. This was the main limitation of this study.

## CONCLUSION

In conclusion, according to HIT diagnosis criteria, we could not define HIT for all the 20 patients. Although the incidence of HIT was low in patients undergoing open heart surgery, an increased rate of early mortality was observed in patients with positive heparin/PF4 antibodies.

**Table:** 

Authors' roles & responsibilities	
ME	Substantial contributions to the conception or design of the work; or the acquisition, analysis, or interpretation of data for the work; drafting the work or revising it critically for important intellectual content; agreement to be accountable for all aspects of the work in ensuring that questions related to the accuracy or integrity of any part of the work are appropriately investigated and resolved; final approval of the version to be published
KTS	Substantial contributions to the conception or design of the work; or the acquisition, analysis, or interpretation of data for the work; final approval of the version to be published
KO	Substantial contributions to the conception or design of the work; or the acquisition, analysis, or interpretation of data for the work; final approval of the version to be published
AS	Drafting the work or revising it critically for important intellectual content; final approval of the version to be published
BA	Drafting the work or revising it critically for important intellectual content; final approval of the version to be published

## References

[1] Nagler M, Bakchoul T (2016). Clinical and laboratory tests for the diagnosis of heparin-induced thrombocytopenia. Thromb Haemost.

[2] Pishko AM, Cuker A (2017). Heparin-induced thrombocytopenia in cardiac surgery patients. Semin Thromb Hemost.

[3] Kerendi F, Thourani VH, Puskas JD, Kilgo PD, Osgood M, Guyton RA (2007). Impact of heparin-induced thrombocytopenia on postoperative outcomes after cardiac surgery. Ann Thorac Surg.

[4] Kuitunen A, Suojaranta-Ylinen R, Raivio P, Kukkonen S, Lassila R (2007). Heparin-induced thrombocytopenia following cardiac surgery is associated with poor outcome. J Cardiothorac Vasc Anesth.

[5] Ortega-Loubon C, Fernández-Molina M, Pañeda-Delgado L, Jorge-Monjas P, Carrascal Y (2018). Predictors of postoperative acute kidney injury after coronary artery bypass graft surgery. Braz J Cardiovasc Surg.

[6] Prince M, Wenham T (2018). Heparin-induced thrombocytopaenia. Postgrad Med J.

[7] Krzych LJ, Nowacka E, Knapik P (2015). Heparin-induced thrombocytopenia. Anaesthesiol Intensive Ther.

[8] Rezende E, Morais G, Silva Junior JM, Oliveira AM, Souza JM, Toledo DO (2011). Thrombocytopenia in cardiac surgery: diagnostic and prognostic importance. Rev Bras Cir Cardiovasc.

[9] Watson H, Davidson S, Keeling D, Haemostasis and Thrombosis Task Force of the British Committee for Standards in Haematology (2012). Guidelines on the diagnosis and management of heparin-induced thrombocytopenia: second edition. Br J Haematol.

[10] Souza RC, Paim L, Viotto G, Aprigio J, Araújo LL, Ribeiro H (2018). Thrombocytopenia after transcatheter valve-in-valve implantation: prognostic marker or mere finding?. Braz J Cardiovasc Surg.

[11] Mujtaba SS, Ledingham S, Shah AR, Schueler S, Clark S, Pillay T (2018). Thrombocytopenia after aortic valve replacement: comparison between sutureless perceval S valve and perimount magna ease bioprosthesis. Braz J Cardiovasc Surg.

[12] Selleng S, Malowsky B, Strobel U, Wessel A, Ittermann T, Wollert HG (2010). Early-onset and persisting thrombocytopenia in post-cardiac surgery patients is rarely due to heparin-induced thrombocytopenia, even when antibody tests are positive. J Thromb Haemost.

[13] Gruel Y, Pouplard C (2010). Post-operative platelet count profile: the most reliable tool for identifying patients with true heparin-induced thrombocypenia after cardiac surgery. J Thromb Haemost.

[14] Patrick AR, Winkelmayer WC, Avorn J, Fischer MA (2007). Strategies for the management of suspected heparin-induced thrombocytopenia: a cost-effectiveness analysis. Pharmacoeconomics.

[15] Pouplard C, May MA, Regina S, Marchand M, Fusciardi J, Gruel Y (2005). Changes in platelet count after cardiac surgery can effectively predict the development of pathogenic heparin-dependent antibodies. Br J Haematol.

[16] Lillo-Le Louët A, Boutouyrie P, Alhenc-Gelas M, Le Beller C, Gautier I, Aiach M (2004). Diagnostic score for heparin-induced thrombocytopenia after cardiopulmonary bypass. J Thromb Haemost.

[17] Lassila R, Antovic JP, Armstrong E, Baghaei F, Dalsgaard-Nielsen J, Hillarp A (2011). Practical viewpoints on the diagnosis and management of heparin-induced thrombocytopenia. Semin Thromb Hemost.

[18] Anna N, Boyan T, Kader MA, Catherine M, Matthias K (2010). The HIT treatment in a cardiac surgery patient. Int J Cardiol.

[19] Linkins LA, Dans AL, Moores LK, Bona R, Davidson BL, Schulman S (2012). Treatment and prevention of heparin-induced thrombocytopenia: antithrombotic therapy and prevention of thrombosis, 9th ed: American college of chest physicians evidence-based clinical practice guidelines. Chest.

[20] Warkentin TE, Sheppard JA, Sigouin CS, Kohlmann T, Eichler P, Greinacher A (2006). Gender imbalance and risk factor interactions in heparin-induced thrombocytopenia. Blood.

[21] Stein PD, Hull RD, Matta F, Yaekoub AY, Liang J (2009). Incidence of thrombocytopenia in hospitalized patients with venous thromboembolism. Am J Med.

[22] Baroletti S, Hurwitz S, Conti NA, Fanikos J, Piazza G, Goldhaber SZ (2012). Thrombosis in suspected heparin-induced thrombocytopenia occurs more often with high antibody levels. Am J Med.

[23] Arangalage D, Lepage L, Faille D, Cimadevilla C, Dilly MP, Papy E (2016). Presentation, management and outcome of heparin-induced thrombocytopenia after valvular heart surgery. Eur J Cardiothorac Surg.

[24] Pathak R, Bhatt VR, Karmacharya P, Aryal MR, Donato AA (2017). Medical and economic burden of heparin-induced thrombocytopenia: a retrospective nationwide inpatient sample (NIS) study. J Hosp Med.

[25] Whitlatch NL, Kong DF, Metjian AD, Arepally GM, Ortel TL Validation of the high-dose heparin confirmatory step for the diagnosis of heparin-induced thrombocytope.

